# Surgical repair of the medial head of the gastrocnemius: two case reports and review

**DOI:** 10.1093/jscr/rjac335

**Published:** 2022-07-16

**Authors:** Vitor Luis Pereira, Carlos Vicente Andreoli, Rafaella Figueiredo Vieira Santos, Paulo Santoro Belangero, Benno Ejnisman, Alberto de Castro Pochini

**Affiliations:** Orthopedics and Traumatology Department, Traumatology Sports Center, Escola Paulista de Medicina, Federal University of São Paulo, São Paulo, SP, Brazil; Orthopedics and Traumatology Department, Traumatology Sports Center, Escola Paulista de Medicina, Federal University of São Paulo, São Paulo, SP, Brazil; Technology and Science University, Salvador, BA, Brazil; Orthopedics and Traumatology Department, Traumatology Sports Center, Escola Paulista de Medicina, Federal University of São Paulo, São Paulo, SP, Brazil; Orthopedics and Traumatology Department, Traumatology Sports Center, Escola Paulista de Medicina, Federal University of São Paulo, São Paulo, SP, Brazil; Orthopedics and Traumatology Department, Traumatology Sports Center, Escola Paulista de Medicina, Federal University of São Paulo, São Paulo, SP, Brazil

## Abstract

The gastrocnemius medial head distal musculotendinous junction injury is relatively common. Musculature contraction in an already stretched structure leads to muscle breakdown. Patients affected are often physically active middle-aged men. The typical presentation includes sudden pain, audible popping, bruising and localized tenderness. Occasionally, there is a palpable defect if the rupture is complete. Although the initial diagnosis can be made on the basis of a careful history and clinical examination, ultrasound or magnetic resonance imaging can be used to better describe the lesion. In complete ruptures, even when conservative treatment shows good results, it is common that the patient presents decreased muscle strength, difficulty returning to sports and permanent and visible gap. Considering surgical treatment in patients with complete ruptures and extensive injuries with a more than 5 cm gap may lead to better healing process, rapid rehabilitation and more efficient return to sports.

## INTRODUCTION

The gastrocnemius medial head distal musculotendinous junction injury, also called ‘tennis leg’ and first described in 1883, is a relatively common clinical condition [1, 2] related as the third most commonly strained muscle in athletes, following the biceps femoris and rectus femoris. It represents the most frequent injury to the posterior compartment of the leg [3, 4]. The gastrocnemius presents a high risk of injury because it crosses two joints (knee and ankle) and has high proportion of fast-twitch muscle fibers (type II), allowing explosions and powerful contractions [1, 5].

Attempting to contract an already stretched muscle can lead to tears, whether during sporting activities or common daily activities, and patients affected are often physically active middle-aged men [1].

The typical presentation is characterized by sudden pain, an audible ‘click’, ecchymosis and localized tenderness in the area of tendinous attachment. Occasionally, there is a palpable defect if the tendon rupture is complete. Although the initial diagnosis can be clinically made, ultrasound or magnetic resonance imaging (MRI) can be used not only for confirmation but also to exclude other diseases and allow the assessment of lesion size. These factors directly influence the choice and duration of treatment; however, the literature review on large size lesions is insufficient and should be improved, so we do not really know if large lesions have the worst prognosis [3, 6].

The surgical approach is rarely described because the basis of treatment is non-surgical [1]. We intend to present our experience in the surgical correction of ruptures of the medial head of the gastrocnemius and the good clinical results we achieved in two patients with extensive lesions. The relative importance, indications for surgical repair and its advantages are also discussed. Both patients agreed to give consent for the publication of their cases.

## CASE REPORT

**Figure 1:**
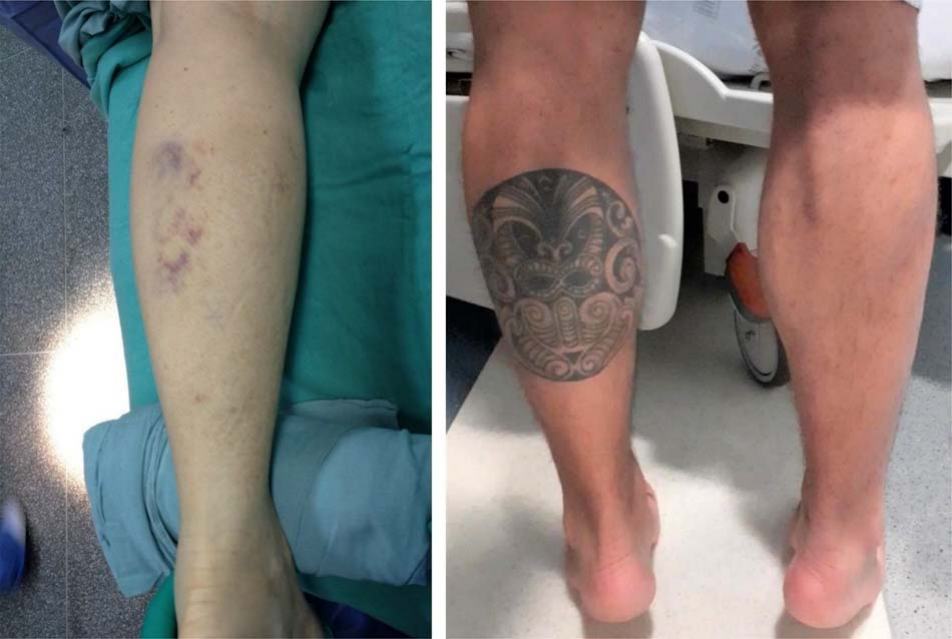
Clinical images of Patient 1 and Patient 2, respectively. In the left image, we observe the presence of posterior ecchymosis on the calf. In the right image, we observe medial muscle space in the left calf associated with significant varus of the hindfoot, previously absent, ipsilateral to the lesion.

Case 1 consists of a 36-year-old female, 10 years amateur volleyball player, who presented with a history of pain and audible popping in the back of the left leg during a jump landing in a volleyball match. She evolved with persistent pain and functional deficit in the leg (Fig. 1A).

Case 2 patient is a practitioner of bodybuilding and competitive amateur cycling, with many years of practice; he intensified training last year. He suffered a fall of about 1.7 m over the bicycle during a journey on rough terrain, and when supporting the right lower limb on the ground, he felt pain and posterior clicking in the right calf, evolving with functional disability in the limb (Fig. 1B).

**Figure 2:**
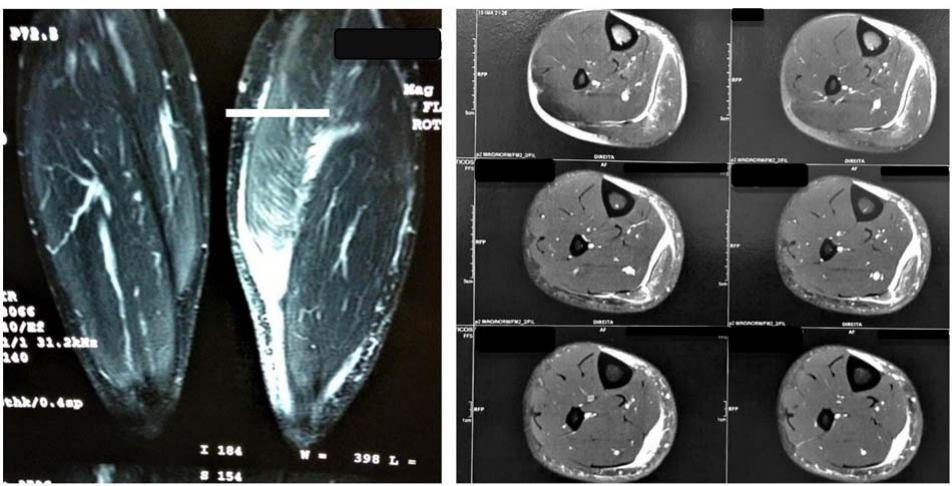
MRI images of Patient 1 and Patient 2, respectively. In the left image, a coronal section of the two legs weighted in a sensitive liquid sequence shows edema accompanied by a distal gap in the lesion topography. In the right image, we see multiple axial slices also showing fluid accumulation and muscle retraction in the musculotendinous transition.

Both patients denied steroids use. They were evaluated by radiographs and MRI and classified by O’Donoghue classification system as a grade III injury in both cases. Lesions were found at the medial head of the gastrocnemius muscle myotendinous transition. In Case 1, 5 cm retraction was measured and in Case 2, 7 cm. Figure 2 shows imaging findings.

Muscle injury were classified by O’Donoghue in three grades according to size and functional loss: grade I for irrelevant tissue lesions, grade II for tissue lesions associated with strength reduction in the muscle-tendon complex and grade III for complete rupture of the muscle-tendon complex and complete functional loss [1, 4, 6].

Due to the presence of significant gap, hematoma and poor improvement with conservative treatment, patients went for surgery. In Case 1, surgery was performed 3 weeks after the trauma, while in Case 2 it was performed after 4 weeks.

**Figure 3:**
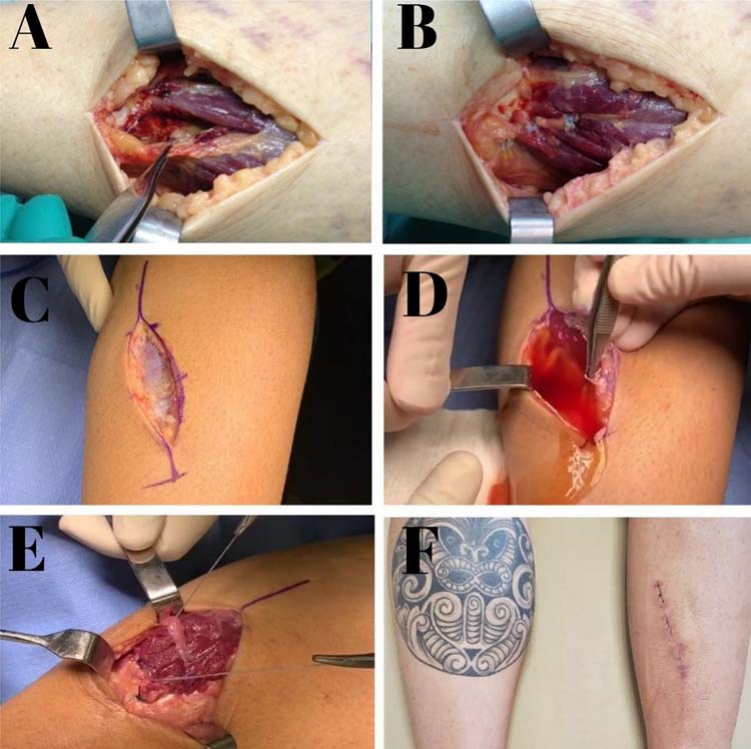
Intraoperative images of Patient 1 (**A**, **B**); intraoperative and postoperative images of Patient 2 (**C**–**F**). (A) Image of a complete gastrocnemius muscle medial head lesion with large gap; (B) suture and complete closure; (C) posteromedial incision above the lesion; (D) abundant outflow of fluid collection after identification of muscle rupture; (E) lesion sutures with total repair of the myotendinous part; (F) aspect of the incision at 3 weeks postoperatively.

A direct posterior incision measuring approximately 7 cm over the medial head of the gastrocnemius was made. Significant gap and scar tissue were observed at the proximal and distal edges of the lesion. Muscle adhesions in the fascia were released; we found abundant drainage of liquid collection, translucent with inflammatory characteristic. After delimitation of the lesion, suture was performed with high-resistance FiberWire® (Arthrex, Naples, FL, USA) and Vycril® number 0.2 threads, with muscular edges fixation and sutures. Two pairs of Krakow anchored stitches sutures, in order to grab the whole muscular mass, were tied. This prevents tears on the muscle mass and gives us a more rigid construct to suture the hole muscle structure. The key points of the described approach are highlighted in Fig. 3.

Both patients evolved with excellent results. They were conducted with temporary suropodalic immobilization on the operated limb without load for 3 weeks. In the next 3 weeks, a physiotherapeutic rehabilitation protocol was started with movement gain, flexibility and gait training. After the sixth week, strengthening and introduction to the sports gesture were started. Case 1 patient returned to sports activities 5 months after the operation and Case 2 patient returned after 4 months and 2 weeks.

## DISCUSSION

Routine tennis leg treatment is conducted non-surgically involving rest, cryotherapy, leg elevation, medication and a physical therapy program. Surgical treatment was reported only in association with compartment syndrome, and rarely the edema is extensive enough to cause it [1, 6, 7].

In cases of total tears, the contracted muscle mass and the gap between the extremities can heal with fibrotic and rigid tissue, bringing an increased risk of injury recurrence, persistent pain and preventing recovery of full strength [1, 8, 9]. Consequently, we believe that the bigger the lesion, the worse healing pattern and following muscle function will become.

Clinically, myotendinous junction injury takes longer than a purely muscular injury to heal and allow return to sports activity, and this is another important treatment issue to consider [9]. Fast return to sports and quality of muscle contraction are important factors in recreational and competitive athletes. Thus, considering surgical treatment in patients with complete tears, extensive injuries and large retractions can lead to better healing process, fast rehabilitation with strength recovery and better clinical outcomes.

Fluid collection between the aponeuroses of the medial head of the gastrocnemius and soleus was seen in ‘tennis leg’ cases, especially at the level of the muscle belly or myotendinous junction, with pseudocystic features [10]. In our study, the presence of collection in this location was a common finding in both patients. We believe that this fluid concentration, which does not have hematoma characteristics, reflects worse injury patterns and worse prognosis for healing. Delgado *et al*. reported this same finding occurring in more than 50% of these patients.

Reviewing the literature, lesions of other muscle groups were also treated surgically with satisfactory reported results. There are no well-established values to consider the gap as large. Based on our clinical experience, we consider 5 cm as an expressive value; however, further studies are needed to validate this observation.

We found only one study addressing surgical treatment. Cheng *et al*. reported two cases, both presenting sports grade III O’Donoghue lesions; a 37-year-old man operated 10 days after injury and a 43-year-old woman operated 10 months after injury. Our study reported similar cases, except for the time of surgery decision. We believe that conservative treatment must be introduced initially, but also can’t be delayed too much; thus, we recommend surgery at 3–4 weeks after injury.

Finally, the authors recommend to consider surgery in young people actively involved in sports with persistent pain, obvious defect and calf extension dysfunction, to ensure strength for continued sports activity [1]. Literature shows that a severe muscle tear treated conservatively will probably heal with fibrotic scar tissue and is thus at an increased risk of recurrent injury [1, 8]. Better anatomical and functional outcomes are expected after surgical repair when compared to conservative treatment [1].

This study demonstrated that patients with gastrocnemius medial head grade III muscle injuries that did do not show improvement after a short period of conservative treatment can be successfully treated by surgical repair and postoperative physical therapy. Nevertheless, as any surgical treatment, we must be caution about potential complications and surgery indications. We recommend future studies to better evaluate this new treatment possibility.

## CONFLICT OF INTEREST STATEMENT

The authors declare no conflict of interest in carrying out this paper.
